# Evaluation of a new method of calculating breast tumor volume based on automated breast ultrasound

**DOI:** 10.3389/fonc.2022.895575

**Published:** 2022-09-13

**Authors:** Jing-Jing Ma, Shan Meng, Sha-Jie Dang, Jia-Zhong Wang, Quan Yuan, Qi Yang, Can-Xu Song

**Affiliations:** ^1^ Department of Internal Medicine, Xi’an Fifth Hospital, Xi’an, China; ^2^ Department of Hematology, The Second Affiliated Hospital of Xi’an Jiaotong University, Xi’an, China; ^3^ Department of Anesthesia, Shaanxi Provincial Cancer Hospital, Affiliated to Xi’an Jiaotong University, Xi’an, China; ^4^ Department of General Surgery, The Second Affiliated Hospital of Xi’an Jiaotong University, Xi’an, China; ^5^ Department of Ultrasound, Shaanxi Provincial Cancer Hospital, Affiliated to Xi’an Jiaotong University, Xi’an, China; ^6^ Department of Surgery, Shaanxi Provincial Cancer Hospital, Affiliated to Xi’an Jiaotong University, Xi’an, China

**Keywords:** breast lesion, volume, automated breast ultrasound system, pixel method, surgery

## Abstract

**Objective:**

To evaluate the effectiveness and advantages of a new method for calculating breast tumor volume based on an automated breast ultrasound system (ABUS).

**Methods:**

A total of 42 patients (18–70 years old) with breast lesions were selected for this study. The Ivenia ABUS 2.0 (General Electric Company, USA) was used, with a probe frequency of 6–15 MHz. Adobe Photoshop CS6 software was used to calculate the pixel ratio of each ABUS image, and to draw an outline of the tumor cross-section. The resulting area (in pixels) was multiplied by the pixel ratio to yield the area of the tumor cross-section. The Wilcoxon signed rank test and Bland-Altman plot were used to compare mean differences and mean values, respectively, between the two methods.

**Results:**

There was no significant difference between the tumor volumes calculated by pixel method as compared to the traditional method (*P*>0.05). Repeated measurements of the same tumor volume were more consistent with the pixel method.

**Conclusion:**

The new pixel method is feasible for measuring breast tumor volume and has good validity and measurement stability.

## Introduction

Breast cancer ranks first in cancer deaths among women. The latest data on global cancer burden (2020) show that breast cancer accounts for 11.7% of all new cancer cases, officially displacing lung cancer as the most prevalent cancer type worldwide (1). In China, there were 420,000 new breast cancer cases, ranking it first in the world (2). Chemotherapy is one of the most commonly prescribed treatment methods for breast cancer (3), and its effectiveness relies heavily on imaging methods to evaluate tumor volume (4).

Various methods have been used to assess tumor volume (5). Magnetic resonance imaging (MRI) has excellent soft tissue resolution, uses no radiation, and offers multi-directional and multi-sequence imaging; however, the examination is time-consuming and expensive (6). Computerized tomography (CT) examination has high spatial and density resolution (7), but uses a large radiation dose and contrast agents with negative side effects. In comparison, ultrasound examination offers the advantages of being affordable, easy to administer, and radiation-free, and is thus recognized as the preferred imaging method for breast cancer (8). It is difficult to achieve accurate measurement of dynamic and complex entities with two-dimensional ultrasound, so volumetric parameters are measured with three-dimensional ultrasound (9, 10).

Automated breast ultrasound (ABUS) is a three-dimensional ultrasound technology for breast examination (11). It uses a standardized, automated imaging system that stores image data and has good repeatability (12). Its unique advantages contribute to its important role in the diagnosis and treatment of breast tumors (13). Currently, only tumor length can be measured on the ABUS system, not cross-sectional area or volume (14). Therefore, ABUS can only estimate volume according to the ellipsoid formula using tumor length, width and height (15). It is clinically necessary to overcome this limitation to ensure accurate and stable measurement of breast tumor volumes (16). Therefore, the authors designed a new method to measure tumor volume using ABUS and evaluated its validity and measurement stability.

## Materials and methods

### General information

This study was approved by the ethics committee of Shaanxi Provincial Cancer Hospital (2021-137) and granted a waiver of informed consent before commencement of the study. The ABUS imaging data of 42 patients with breast tumors who underwent ABUS examination in our hospital from June 2018 to June 2021 were retrospectively analyzed. The validity and measurement stability of the pixel method were compared with those of the traditional method (length × width × height/2). Inclusion criteria: 1) female patients; 2) aged 18–70 years old; 3) with breast tumors - if there were multiple breast tumors, the one with the largest length and diameter was selected; 4) the breast tumor had a well-defined boundary; 5) the long diameter of the tumor was ≥1cm and ≤5 cm. Exclusion criteria: 1) age < 18 years old or > 70 years old; 2) breast tumor with ill-defined boundary; 3) the long diameter of breast tumor was <1cm or >5 cm.

### Instrument and ABUS inspection process

The Invenia ABUS 2.0 (General Electric Company, USA) was used, with a probe frequency of 6–15 MHz, field of view of 15.3 cm, scanning length of 16.9 cm, and maximum scanning depth of 5.0 cm. The patient was instructed to lie in a supine position and breathe calmly. Preset scanning conditions were selected on the instrument according to the size of the patient’s breast. Lateral, medial, and anteroposterior scans were performed bilaterally for all patients, and upper and lower scans were added for larger breasts. After the scan the images were imported into the image viewing system that comes with ABUS for 3D reconstruction, and transverse, sagittal, and coronal cross-sections were obtained.

### Tumor volume measurement

ABUS imaging data of breast tumor volume was evaluated by two physicians, each using both the pixel method and the traditional method. The traditional method is to measure the length, width, and height of the tumor using the image viewing system that comes with ABUS. Using an ABUS coronal image, the length was defined as the largest diameter of the tumor and the width was defined as the largest diameter perpendicular to the length. Using an ABUS cross-section, the height was defined as the largest diameter of the tumor perpendicular to the plane of the ABUS probe on the cross-section was selected as the height (equivalent to the anteroposterior diameter of the tumor *in vivo*). The volume of the tumor was calculated by length*width*height/2. For the pixel method, first the pixel ratio was calculated according to the scale of the original image, and then the tumor was outlined in order to obtain the number of pixels in each cross-section. This process is done in Adobe Photoshop CS6 software. Tumor cross-section area was then calculated by multiplying pixel ratio by the number of pixels in each cross-section. On ABUS images, each breast tumor was divided on the coronal plane at 0.1-cm intervals. For each layer, the area was measured by pixel method, and volume was calculated by multiplying area by height (the height of each layer was 0.1 cm). The total tumor volume was calculated as the sum of the volumes of each layer. Consistency and stability of the two methods were then compared.

### Statistical methods

SPSS 26.0 software was used to perform all statistical analyses. All measurement data were expressed as mean ± SD, and Bland-Altman plots or Wilcoxon signed rank test were used to compare the mean values and differences of the two methods. *P* < 0.05 was considered statistically significant.

## Results

The 42 patients studied ranged from 19 to 65 years old, with an average age of 37.3 ± 12.6 years old. Tumor lengths ranged from 1.0 to 4.9 cm with an average length of 2.17 cm.

**Figure 1:**
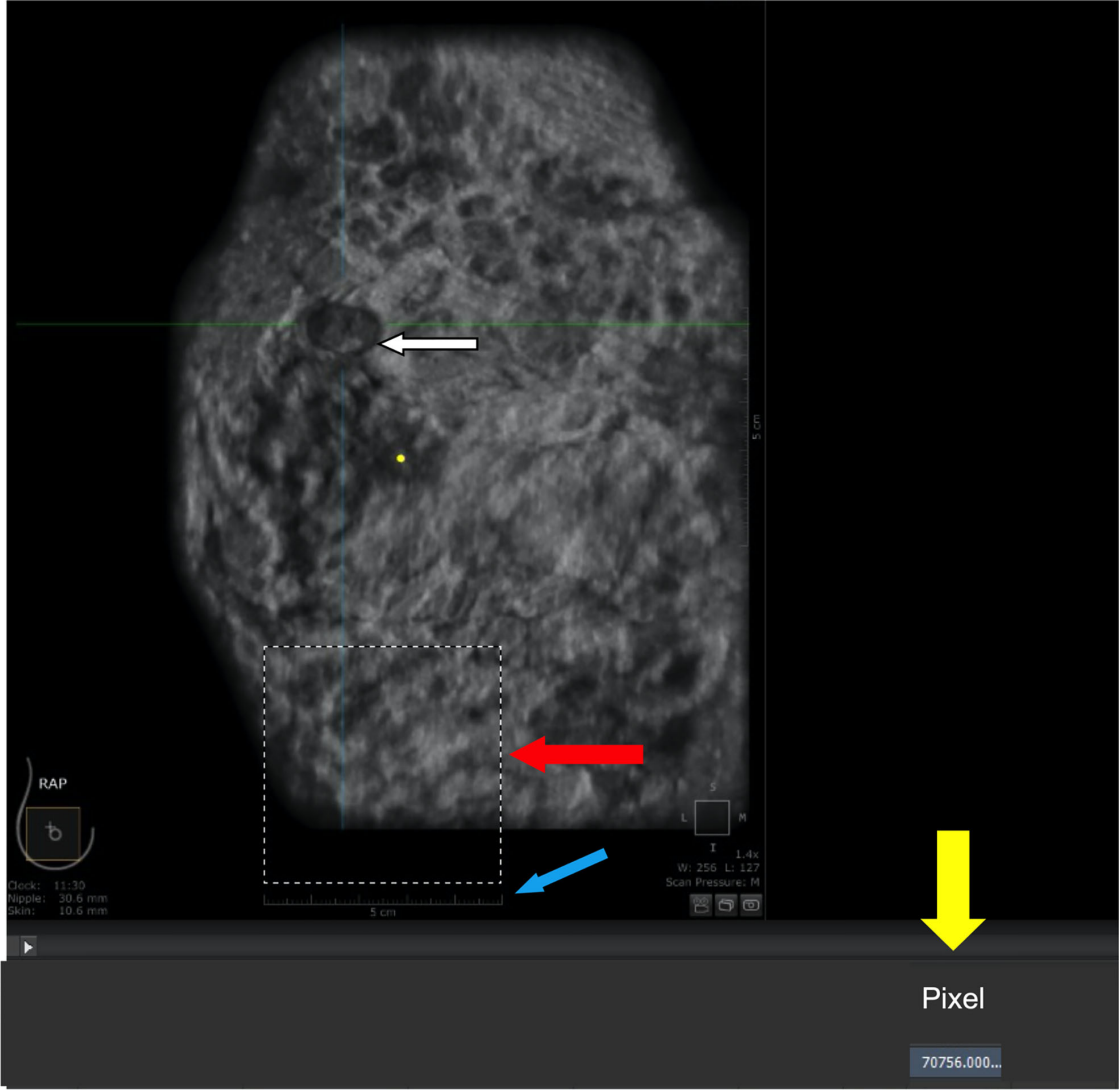
Calculation of area-to-pixel ratio using Adobe Photoshop CS6 software. **(A)** scale bar (5 cm). **(B)** square with a length of 5 cm and actual area of 25 cm^2^. **(C)** number of pixels (70756) automatically counted by the software within the outlined region. **(D)** tumor.

**Figure 2:**
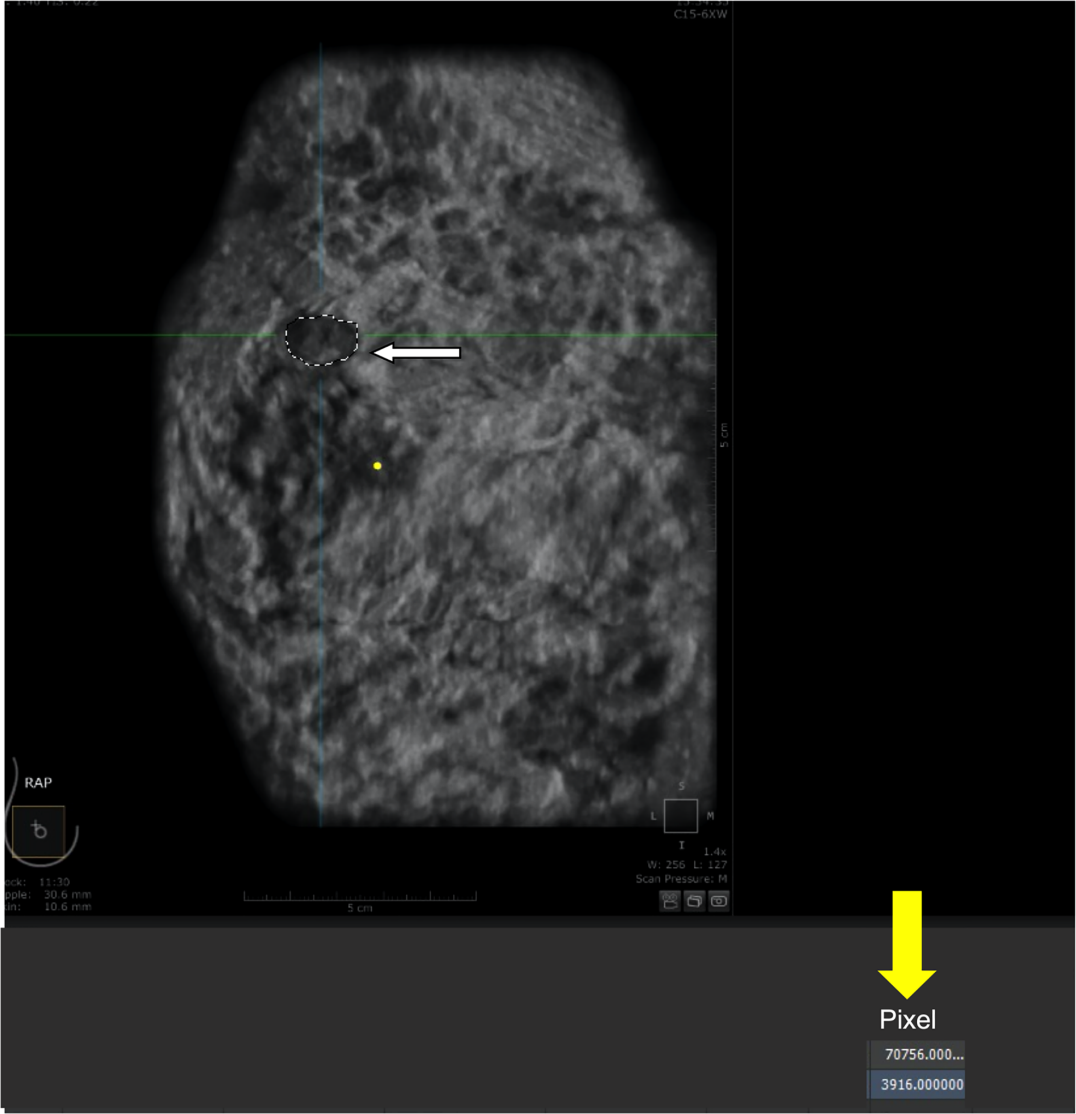
Calculation of pixels within the tumor. **(A)** tumor (outlined by white dashed line). **(B)** number of pixels (3916) automatically counted by the software within the outlined region.

**Figure 3:**
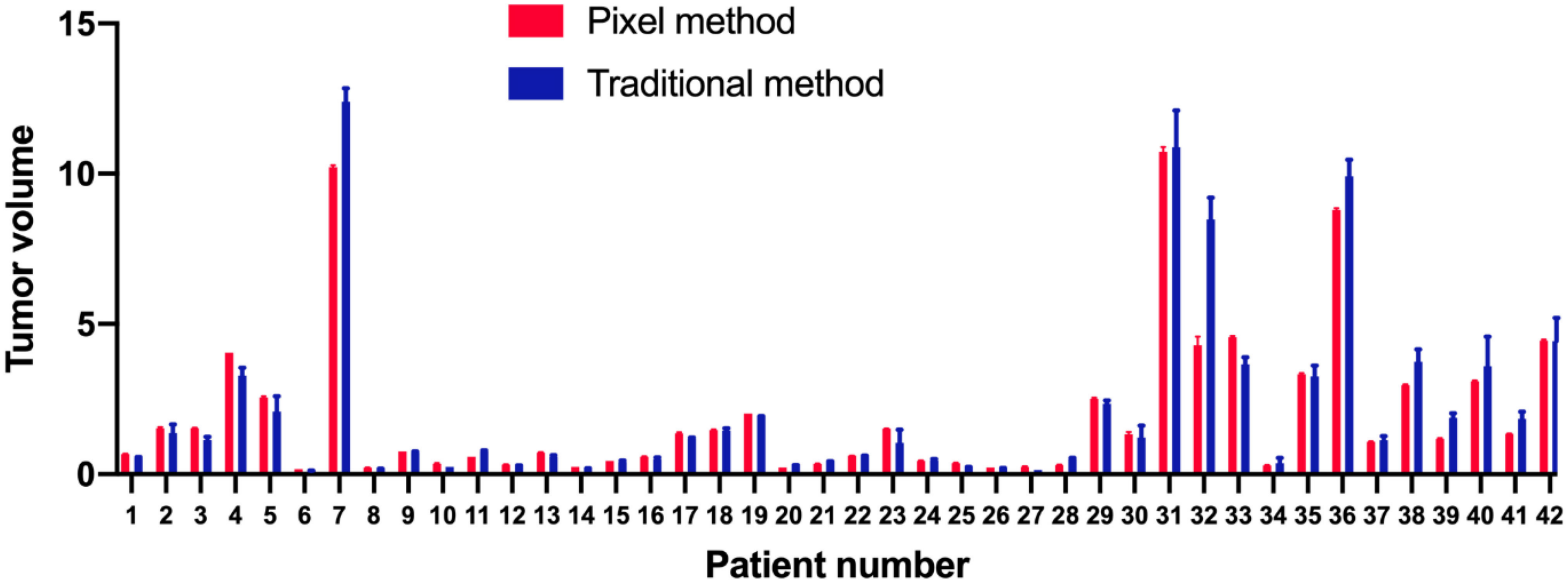
Means and standard deviations of each tumor volume as measured by pixel (red) and traditional (blue) methods.

The analysis of tumor pixels is shown in **Figures 1**, **2**. Coronal images were imported into Adobe Photoshop CS6 software, and the area-to-pixel ratio was calculated according to the scale on the image. **Figure 3** shows the mean volume of each tumor as measured by both pixel and traditional methods.

Patients’ ages and tumor lengths are shown in **Table 1**, along with the differences in tumor volumes as measured by two physicians using both pixel and traditional methods. The mean volume of 42 lesions measured by the pixel method was 1.073 times that measured by the traditional method (standard deviation: 0.266, 95% confidence interval: 0.541–1.605). There was no significant difference in the volume measured by the pixel method as compared to that measured by the traditional method, indicating that the pixel method is feasible and effective for tumor volume measurement (signed rank test, *P*=0.542). The stability of the two methods was assessed by comparing the mean difference between physicians. The mean difference for the pixel method was significantly less than that of the traditional method (*P*<0.01).

**Table 1:** Patient age and tumor length, and difference in tumor volumes measured by two physicians using either the pixel or traditional method.

Patient number	Age	Tumor length (cm)	Tumor volume (cm^3^, Pixel method)		Difference (Pixel method)	Tumor volume (cm^3^, Traditional method)		Difference (Traditional method)
			Doctor1	Doctor2		Doctor1	Doctor2	
1	46	1.8	0.676	0.665	0.011	0.531	0.560	0.029
2	31	2.5	1.503	1.558	0.055	1.152	1.574	0.422
3	30	2.5	1.526	1.541	0.015	1.210	1.059	0.151
4	20	2.4	4.040	4.040	0.000	3.466	3.091	0.375
5	19	2.1	2.535	2.580	0.045	1.716	2.443	0.727
6	19	1.4	0.171	0.172	0.001	0.117	0.102	0.015
7	46	3.3	10.174	10.261	0.087	12.078	12.708	0.630
8	36	1.0	0.218	0.208	0.010	0.177	0.165	0.012
9	29	2.2	0.758	0.760	0.002	0.692	0.750	0.058
10	40	1.0	0.330	0.358	0.028	0.246	0.243	0.003
11	32	1.6	0.582	0.578	0.004	0.788	0.733	0.055
12	51	1.2	0.320	0.325	0.005	0.263	0.290	0.027
13	37	1.8	0.730	0.719	0.011	0.552	0.619	0.067
14	28	1.4	0.246	0.245	0.001	0.165	0.184	0.019
15	41	1.6	0.444	0.441	0.003	0.367	0.436	0.069
16	65	1.3	0.571	0.586	0.015	0.444	0.542	0.098
17	35	1.8	1.384	1.328	0.056	1.154	1.211	0.057
18	25	2.2	1.487	1.451	0.036	1.403	1.505	0.102
19	51	2.6	2.017	2.022	0.005	1.926	1.905	0.021
20	37	1.3	0.228	0.227	0.001	0.275	0.302	0.027
21	21	1.2	0.347	0.329	0.018	0.421	0.388	0.033
22	28	1.3	0.619	0.610	0.009	0.611	0.569	0.042
23	18	2.3	1.508	1.518	0.010	0.724	1.355	0.631
24	36	1.9	0.447	0.449	0.002	0.431	0.490	0.059
25	45	1.4	0.362	0.376	0.014	0.227	0.243	0.016
26	55	1.2	0.232	0.232	0.000	0.174	0.204	0.030
27	27	1.1	0.225	0.260	0.035	0.148	0.147	0.001
28	25	1.4	0.301	0.311	0.010	0.479	0.534	0.055
29	37	2.9	2.538	2.483	0.055	2.257	2.418	0.161
30	47	1.8	1.286	1.384	0.098	0.931	1.496	0.565
31	25	4.9	10.606	10.848	0.242	10.023	11.749	1.726
32	57	4.1	4.501	4.099	0.402	8.992	7.965	1.027
33	28	2.8	4.550	4.598	0.048	3.480	3.825	0.345
34	26	1.9	0.290	0.302	0.012	0.252	0.496	0.244
35	59	3.1	3.299	3.358	0.059	3.512	3.009	0.503
36	52	4.4	8.838	8.748	0.090	9.523	10.309	0.786
37	32	2.0	1.077	1.090	0.013	1.228	1.043	0.185
38	25	3.8	2.954	2.984	0.030	3.456	4.033	0.577
39	59	2.9	1.164	1.198	0.034	1.774	1.983	0.209
40	55	2.6	3.114	3.076	0.038	2.891	4.288	1.397
41	27	2.2	1.351	1.344	0.007	1.665	2.013	0.348
42	51	3.0	4.466	4.421	0.045	3.889	4.980	1.091

difference values were compared using the Wilcoxon signed rank test (W_-_=879, W_+_=24, T_0.01(42)_ =247-656, P<0.01).

**Figure 4:**
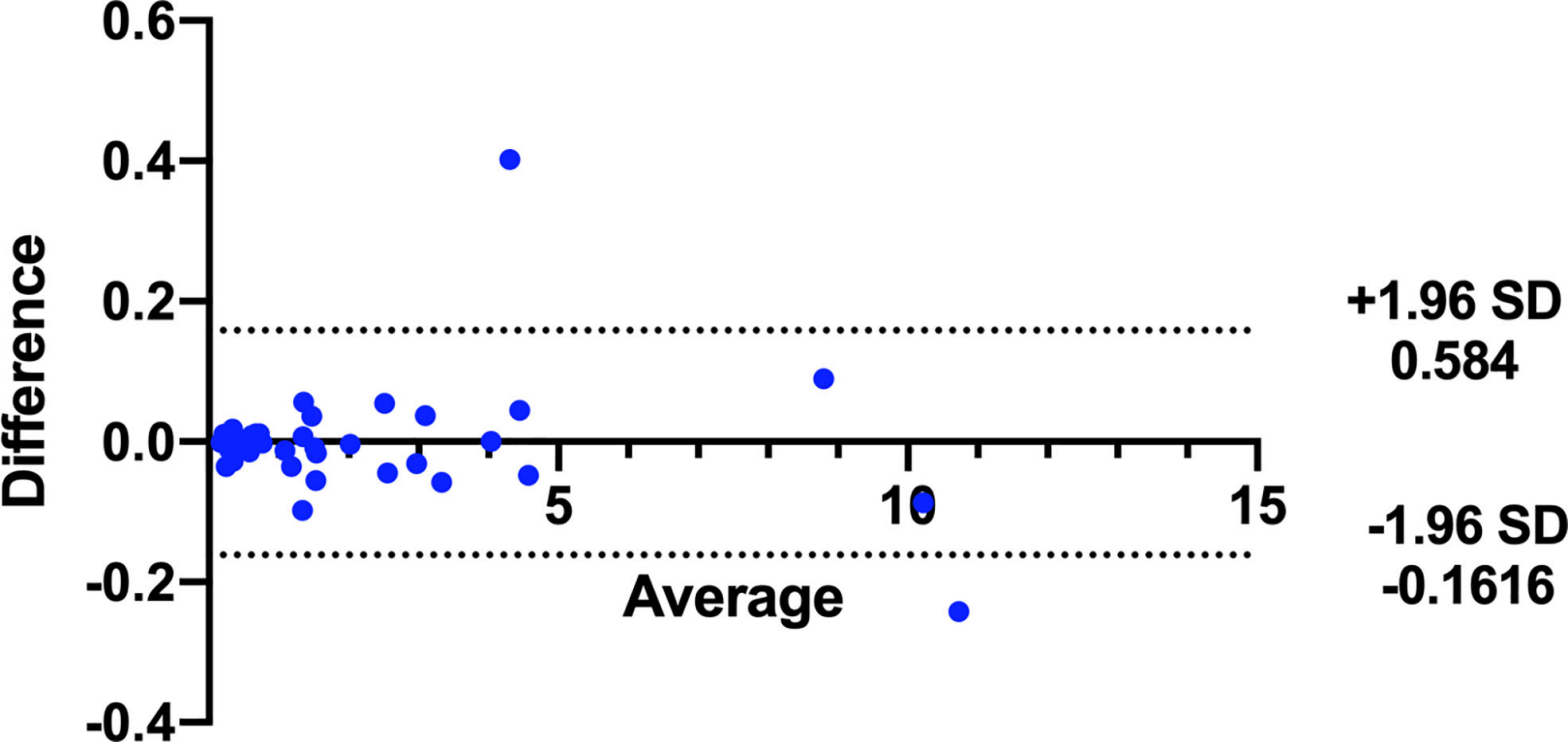
Bland-Altman analysis of tumor volumes measured by two doctors using the pixel method.

**Figure 5:**
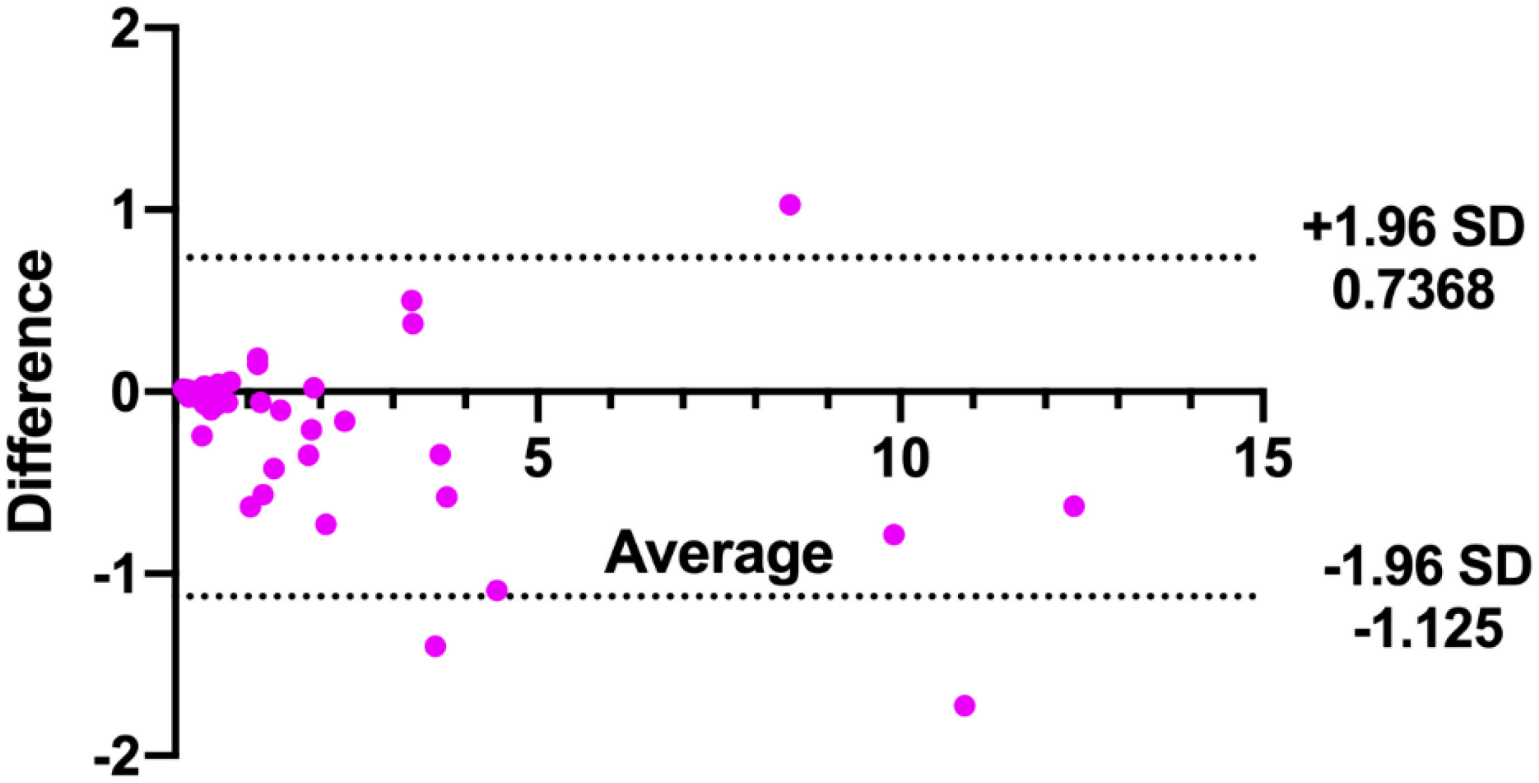
Bland-Altman analysis of tumor volumes measured by two doctors using the traditional method.

In addition, the consistency of the two physicians’ measurements was assessed using Bland-Altman analysis. As shown in **Figure 4**, the majority of tumor volumes measured by the pixel method were within the 95% confidence range. In contrast, more tumor volumes measured by the traditional method fell outside the 95% confidence range (shown in **Figure 5**), further illustrating the higher consistency of the pixel method. The mean and standard deviation of the difference between the measured values of the two physician based on the pixel method was smaller, and there was no statistical difference between the measured values of the two doctors (signed rank test, P=0.300). In contrast, there was a statistically significant difference between the two doctors’ measurement values using the traditional method (signed rank test, P=0.001) (**Table 2**). These results show that the pixel method has smaller measurement errors, more stable results, and less subjective influence by physician.

**Table 2:** Comparison between the difference of tumor volumes measured by two physicians using either the pixel or traditional method.

	Pixel method		Traditional method	
	Difference	P	Difference	P
Mean (SD)	-0.0016 (0.0816)	0.300	-0.194 (0.475)	0.001
median (Q1, Q3)	-0.002 (-0.028,0.010)		-0.058 (-0.348,0.012)	

difference values were compared using the signed rank test.

## Discussion

In this study, a new pixel method was developed to measure the volume of breast tumors based on ABUS imaging. The results demonstrate that compared with the traditional method, the pixel method offers less error and more stability in measuring the volume of breast tumors.

ABUS uses a standardized, automated system for the acquisition and storage of image data (13, 17). Tomographic images similar to MRI and CT can be obtained in transverse, sagittal, and coronal planes, providing sufficient information from which to calculate target volume (18, 19). However, the current ABUS system can only measure tumor length, and has not yet been used to measure cross-sectional area or volume. The present study used pixel ratios to calculate cross-sectional areas and whole-tumor volumes from ABUS images. Results indicated that tumor volumes calculated by the pixel method were slightly higher than, but not significantly different from, those calculated by the traditional method. This indicates that the performance of the pixel method is comparable to that of the traditional method. Bland-Altman analysis showed that more tumor volumes measured by the pixel method fell within the 95% confidence range as compared to those measured by the traditional method, further illustrating the higher consistency of the two physicians’ measurements by the pixel method. The ultimate goal of the pixel method is to enable automated measurement of tumor volumes by a computer.

Not only does the pixel method offer smaller error and greater measurement stability, but it can also measure tumors with irregular shapes. However, one disadvantage is that the measurement time is long. In this study, the time required to calculate the breast tumor volume of each patient was about 2-10 minutes. If the method were to be merged into existing clinical software, rather than having to import to Photoshop, then the calculation time would be significantly reduced (20). In addition, the present study only considered tumors with well-defined boundaries. Many malignant breast lesions are ill-defined, and whether the pixel method would be suitable for them requires further investigation.

In conclusion, the pixel method is feasible and effective to measure the volume of breast tumors, with small error and good stability of the measured value.

## Data availability statement

The raw data supporting the conclusions of this article will be made available by the authors, without undue reservation.

## Ethics statement

This study was approved by the ethics committee of Shaanxi Provincial Cancer Hospital (2021-137). The ethics committee waived the requirement of written informed consent for participation.

## Author contributions

J-JM and SM contributed to the manuscript writing. S-JD and J-ZW conducted literature research and enrolled the participants. QYa and QYu contributed to the data acquisition and analysis. C-XS contributed to the study design and revised the manuscript. All authors contributed to the article and approved the submitted version. All authors agree to be accountable for all aspects of the work in ensuring that questions related to the accuracy or integrity of the work are appropriately investigated and resolved.

## Funding

This work was supported by scientific research project of Shaanxi Provincial Cancer Hospital (No. SXZL-2021-001A).

## Acknowledgments

We thank Medjaden Inc. for scientific editing of this manuscript.

## Conflict of interest

The authors declare that the research was conducted in the absence of any commercial or financial relationships that could be construed as a potential conflict of interest.

## Publisher’s note

All claims expressed in this article are solely those of the authors and do not necessarily represent those of their affiliated organizations, or those of the publisher, the editors and the reviewers. Any product that may be evaluated in this article, or claim that may be made by its manufacturer, is not guaranteed or endorsed by the publisher.

## References

[1] Sung H Ferlay J Siegel RL Laversanne M Soerjomataram I Jemal A. Global cancer statistics 2020: GLOBOCAN estimates of incidence and mortality worldwide for 36 cancers in 185 countries. CA Cancer J Clin (2021) 71:209–49. doi: 10.3322/caac.21660 33538338

[2] Xia C Dong X Li H Cao M Sun D He S. Cancer statistics in China and united states, 2022: profiles, trends, and determinants. Chin Med J (Engl) (2022) 135:584–90. doi: 10.1097/cm9.0000000000002108 PMC8920425 35143424

[3] Alkabban FM Ferguson T. Breast cancer. In: StatPearls. Treasure Island (FL: StatPearls Publishing (2021). 29493913

[4] Tripathy D Im SA Colleoni M Franke F Bardia A Harbeck N. Ribociclib plus endocrine therapy for premenopausal women with hormone-receptor-positive, advanced breast cancer (MONALEESA-7): a randomised phase 3 trial. Lancet Oncol (2018) 19:904–15. doi: 10.1016/s1470-2045(18)30292-4 29804902

[5] Youn I Choi S Choi YJ Moon JH Park HJ Ham SY. Contrast enhanced digital mammography versus magnetic resonance imaging for accurate measurement of the size of breast cancer. Br J Radiol (2019) 92:20180929. doi: 10.1259/bjr.20180929 31017460 PMC6592089

[6] Li H Yao L Jin P Hu L Li X Guo T. MRI And PET/CT for evaluation of the pathological response to neoadjuvant chemotherapy in breast cancer: A systematic review and meta-analysis. Breast (2018) 40:106–15. doi: 10.1016/j.breast.2018.04.018 29758503

[7] Li J Gao W Yu B Wang F Wang L. Multi-slice spiral CT evaluation of breast cancer chemotherapy and correlation between CT results and breast cancerspecific gene 1. J Buon (2018) 23:378–83. 29745080

[8] Dobruch-Sobczak K Piotrzkowska-Wróblewska H Klimonda Z Roszkowska-Purska K Litniewski J. Ultrasound echogenicity reveals the response of breast cancer to chemotherapy. Clin Imaging (2019) 55:41–6. doi: 10.1016/j.clinimag.2019.01.021 30739033

[9] Vourtsis A. Three-dimensional automated breast ultrasound: Technical aspects and first results. Diagn Interv Imaging (2019) 100:579–92. doi: 10.1016/j.diii.2019.03.012 30962169

[10] Schmachtenberg C Fischer T Hamm B Bick U. Diagnostic performance of automated breast volume scanning (ABVS) compared to handheld ultrasonography with breast MRI as the gold standard. Acad Radiol (2017) 24:954–61. doi: 10.1016/j.acra.2017.01.021 28336007

[11] Depretto C Liguori A Primolevo A Di Cosimo S Cartia F Ferranti C. Automated breast ultrasound compared to hand-held ultrasound in surveillance after breast-conserving surgery. Tumori (2021) 107:132–8. doi: 10.1177/0300891620930278 32552398

[12] Vourtsis A Kachulis A. The performance of 3D ABUS versus HHUS in the visualisation and BI-RADS characterisation of breast lesions in a large cohort of 1,886 women. Eur Radiol (2018) 28:592–601. doi: 10.1007/s00330-017-5011-9 28828640

[13] Chiang TC Huang YS Chen RT Huang CS Chang RF. Tumor detection in automated breast ultrasound using 3-d CNN and prioritized candidate aggregation. IEEE Trans Med Imaging (2019) 38:240–9. doi: 10.1109/tmi.2018.2860257 30059297

[14] Zhang P Ma Z Zhang Y Chen X Wang G. Improved inception V3 method and its effect on radiologists’ performance of tumor classification with automated breast ultrasound system. Gland Surg (2021) 10:2232–45. doi: 10.21037/gs-21-328 PMC8340346 34422594

[15] Li Y Wu W Chen H Cheng L Wang S. 3D tumor detection in automated breast ultrasound using deep convolutional neural network. Med Phys (2020) 47:5669–80. doi: 10.1002/mp.14477 32970838

[16] Lacson R Goodrich ME Harris K Brawarsky P Haas JS. Assessing inaccuracies in automated information extraction of breast imaging findings. J Digit Imaging (2017) 30:228–33. doi: 10.1007/s10278-016-9927-4 PMC5359211 27844217

[17] Lee CY Chang TF Chou YH Yang KC. Fully automated lesion segmentation and visualization in automated whole breast ultrasound (ABUS) images. Quant Imaging Med Surg (2020) 10:568–84. doi: 10.21037/qims.2020.01.12 PMC7136742 32269918

[18] Lagendijk M Vos EL Ramlakhan KP Verhoef C Koning AHJ van Lankeren W. Breast and tumour volume measurements in breast cancer patients using 3-d automated breast volume scanner images. World J Surg (2018) 42:2087–93. doi: 10.1007/s00268-017-4432-6 PMC5990576 29299647

[19] Huang A Zhu L Tan Y Liu J Xiang J Zhu Q. Evaluation of automated breast volume scanner for breast conservation surgery in ductal carcinoma in situ. Oncol Lett (2016) 12:2481–4. doi: 10.3892/ol.2016.4924 PMC5038336 27698816

[20] Moon WK Huang YS Hsu CH Chang Chien TY Chang JM Lee SH. Computer-aided tumor detection in automated breast ultrasound using a 3-d convolutional neural network. Comput Methods Programs BioMed (2020) 190:105360. doi: 10.1016/j.cmpb.2020.105360 32007838

